# The Relationship Between Anthropometric Variables and Lung Function in a Severe Smoking Community Population With Ventilatory Dysfunction

**DOI:** 10.1111/crj.70076

**Published:** 2025-05-28

**Authors:** Tiantian Cen, Zekai Cen, Xuan Chen, Zaichun Deng, Yiming Yu, Shanshan Wang, Hongying Ma

**Affiliations:** ^1^ Department of Respiratory and Critical Care Medicine, Key Laboratory of Respiratory Disease of Ningbo The First Affiliated Hospital of Ningbo University Ningbo Zhejiang China

**Keywords:** body mass index, chronic obstructive pulmonary disease, chronic obstructive pulmonary disease population screener, chronic obstructive pulmonary disease screening questionnaire, lung function, waist circumference

## Abstract

**Background:**

The World Health Organization estimated that 65 million individuals have chronic obstructive pulmonary disease (COPD). However, large numbers remain undiagnosed. Anthropometric variables and lung function are closely related, such as body mass index (BMI), waist circumference (WC), and waist‐to‐height ratio (WHtR). Therefore, it is essential to explore the relationship between anthropometric variables and lung function.

**Methods:**

We recruited 7679 severe smokers. Severe smoking was defined as a smoking index ≥ 20 pack‐years. Among these participants, there are 6214 severe smokers with mild, moderate, and moderately severe obstructive ventilation dysfunction and 1465 severe smokers with severe and very severe obstructive ventilation dysfunction. Otherwise, participants were divided into different groups according to questionnaires and sex.

**Results:**

Lung function in the severe smoking community population was associated with anthropometric variables. The study results showed that BMI was negatively associated with the risk of severe and very severe obstructive ventilation dysfunction in a severe smoking community population with ventilatory dysfunction (OR 0.791, 95% CI 0.691–0.907, *p* = 0.001), the COPD Population Screener (COPD‐PS) scores ≥ 5 group (OR 0.787, 95% CI 0.688–0.902, *p* = 0.001), the COPD Screening Questionnaire (COPD‐SQ) scores ≥ 16 group (OR 0.791, 95% CI 0.689–0.908, *p* = 0.001), the COPD‐PS scores ≥ 5 and COPD‐SQ scores ≥ 16 group (OR 0.730, 95% CI 0.603–0.884, *p* = 0.001) and the male group (OR 0.813, 95% CI 0.708–0.933, *p* = 0.003). The study showed that WC was also associated with obstructive ventilation dysfunction.

**Conclusion:**

Low BMI and WC were independent risk factors for severe and very severe obstructive ventilation dysfunction in the severe smoking community Chinese population with ventilatory dysfunction. Collecting COPD questionnaires may help manage lung function in the community population.

## Graphical abstract

BMI was an independent risk factor for obstructive ventilation dysfunction. WC was an independent risk factor for obstructive ventilation dysfunction. Questionnaires help manage lung function and anthropometric variables.

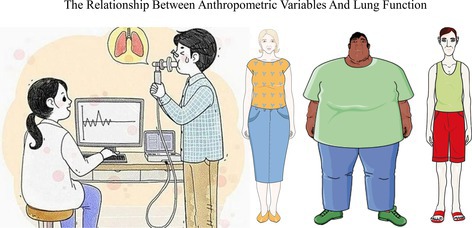

## 1 Introduction

Chronic obstructive pulmonary disease (COPD) is a heterogeneous lung condition characterized by chronic respiratory symptoms (dyspnea, cough, sputum production) due to abnormalities of the airways (bronchitis, bronchiolitis) and/or alveoli (emphysema) that cause persistent, often progressive, airflow obstruction. There are lots of COPD questionnaires, including COPD‐PS and COPD Screening Questionnaire (COPD‐SQ). The COPD‐PS and COPD‐SQ had comparable discriminatory power for detecting COPD in the general population. Both COPD‐PS scores ≥ 5 and COPD‐SQ scores ≥ 16 have high diagnostic values for COPD.

There are some anthropometric measures, including body mass index (BMI), waist circumference (WC), waist body mass index (wBMI), and waist‐to‐height ratio (WHtR). These indicators can measure the degree of body fat and thinness in different dimensions and have been previously proposed to predict abnormal cardiac geometry, insulin resistance, increased arterial stiffness, and dyslipidemia.

According to ATS/ERS, the grade of ventilatory dysfunction was defined as the percentage value of forced expiratory volume in 1 s predicted (FEV1%), regardless of obstructive, restrictive, or mixed ventilatory impairment. Mild ventilatory dysfunction was defined as FEV1% ≥ 70% but greater than lower limits of normal (LLNs) or the ratio of forced expiratory volume in 1 s to forced vital capacity (FEV1/FVC) < LLN; moderate ventilatory dysfunction was defined as an FEV1% of 60%–69%; moderate‐to‐severe ventilatory dysfunction was defined as an FEV1% of 50%–59%; severe ventilatory dysfunction was defined as an FEV1% of 35%–49%; and very severe ventilatory dysfunction was defined as FEV1% less than 35% [1]. Airflow limitation can occur in a variety of respiratory diseases. COPD is closely related to ventilatory dysfunction, and it is mainly characterized by obstructive ventilation dysfunction. In the late stage of COPD, some patients may be combined with restrictive pulmonary ventilation dysfunction and manifest as mixed ventilation dysfunction.

Smoking can increase the risk of lung cancer, heart problems, and stroke. It can also cause smoking‐related lung diseases. Although it is essential to expand the taxonomy (classification) of COPD to include non–smoking‐related COPD types, smoking remains the leading cause of COPD. Severe smoking can affect health, especially the respiratory system. Smoking is common in the community population. According to China's reported health hazards of smoking in 2020, smoking can lead to COPD, and the longer the smoking history, the higher the risk of COPD. However, COPD caused by smoking is not managed well in the community, like hypertension and diabetes.

Our study aimed to explore the relationship between anthropometric variables and lung function in the severe smoking community population, specifically examining the severe smoking community population at risk of COPD.

## 2 Methods

### 2.1 Study Design and Patients

The severity of any spirometric abnormality is based on the forced expiratory volume in 1 s (FEV1) according to the European Respiratory Society: Mild obstructive ventilation dysfunction was defined as FEV1% ≥ 70% and < LLN; moderate obstructive ventilation dysfunction was defined as FEV1% ≥ 60% and < 70%; moderately severe obstructive ventilation dysfunction was defined as FEV1% ≥ 50% and < 60%; severe obstructive ventilation dysfunction was defined as FEV1% ≥ 35% and < 50%; and very severe obstructive ventilation dysfunction was defined as FEV1% < 35%. All operators were trained and standardized.

Severe smoking was defined as a smoking index ≥ 20 pack‐years [2]. The smoking index is the product of the number of cigarettes smoked per year and the number of years of smoking.

COPD‐PS, designed by Fernando, is a simple COPD screening tool. It is simple in design and contains only five items, with higher scores indicating a greater likelihood of airflow limitation. COPD‐SQ was designed by Zhou et al. [3]. Based on the data of 18 900 subjects from community sources, another group of subjects was used as the validation set to test the prediction effect. The COPD‐SQ includes seven items with a total score of 38.

**FIGURE 1:**
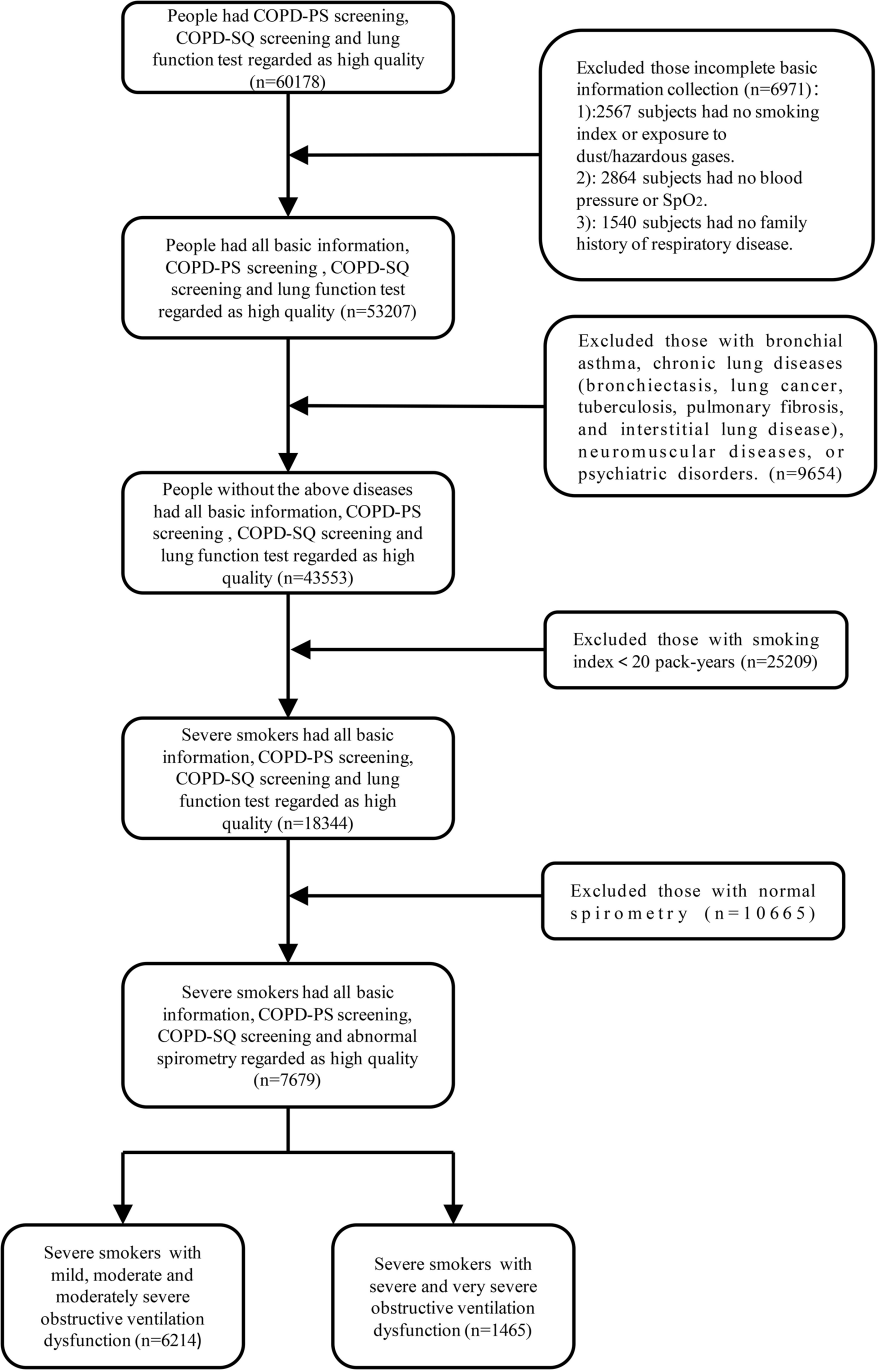
Inclusion and exclusion criteria for severe smokers. COPD‐PS, COPD population screening; COPD‐SQ, COPD screening questionnaire.

In total, 7976 severe smokers with ventilatory dysfunction were enrolled in the Ningbo community between May 2022 and April 2023. The data were from the 2022 Free COPD Screening for Key Populations in Ningbo Program (2022ZYC‐Z31). Among them, 6214 individuals with mild, moderate, and moderately severe obstructive ventilation dysfunction and 1465 individuals with severe and very severe obstructive ventilation dysfunction were enrolled (Figure 1). Among individuals with mild, moderate, and moderately severe obstructive ventilation dysfunction, there were 2347 participants with COPD‐PS scores ≥ 5, 6070 participants with COPD‐SQ scores ≥ 16, 2205 participants with COPD‐PS scores ≥ 5 and COPD‐SQ scores ≥ 16, and 6103 male participants. Among individuals with severe and very severe obstructive ventilation dysfunction, there were 729 participants with COPD‐PS scores ≥ 5, 1446 participants with COPD‐SQ scores ≥ 16, 710 participants with COPD‐PS scores ≥ 5 and COPD‐SQ scores ≥ 16, and 1454 male participants.

For the exclusion criteria, participants with a preexisting diagnosis of bronchial asthma or a history of chronic lung disease (bronchiectasis, lung cancer, tuberculosis, pulmonary fibrosis, and interstitial lung disease), neuromuscular disease, or psychiatric illness were excluded.

The First Affiliated Hospital of Ningbo University's ethical committee granted consent for this study (approval number KY20221208). We attest that the study considered the Helsinki Declaration of 1964 and any subsequent changes. WC is calculated as waist circumference divided by height. BMI is calculated as weight (in kilograms)/height squared (in square meters). wBMI is calculated as WC in meters × BMI in kilograms/square meter, resulting in kilograms per meter.

### 2.2 Statistical Analysis

First, we selected the population that met the inclusion and exclusion criteria. Then, we performed a five‐part analysis. We selected those with COPD‐PS scores ≥ 5, those with COPD‐SQ scores ≥ 16, those with COPD‐PS scores ≥ 5 and COPD‐SQ scores ≥ 16 and the male population from the total population. We also divided them into two groups based on lung function: people with mild, moderate, and moderately severe obstructive ventilation dysfunction and those with severe and severe obstructive ventilation dysfunction.

The specific grouping rules have been described in the previous section. Covariates in the models included age (years), sex (male or female), smoking index (pack‐years), exposure to dust/hazardous gases (yes or no), frequent cough (yes or no), breathlessness (yes or no), systolic blood pressure (mmHg), diastolic blood pressure (mmHg), SpO_2_ (%), resting heart rate (times per minute), family history of respiratory disease (yes or no), COPD‐PS (score), COPD‐SQ (score), FEV1 (L), FVC (L), and FEV1/FVC (%). Significant differences between populations were from univariate analyses to evaluate patient characteristics, and variables that had two‐sided *p*‐values < 0.05 in the univariate analyses and anthropometric variables were included in the multivariate logistic regression analysis. Multivariate logistic regression models analyzed the association between lung function and anthropometric variables. Continuous variables were presented as the mean and standard deviation (SD) when normally distributed. Categorical data were expressed as proportions. *T*‐test analysis of variance was used to compare the differences in anthropometric variables between two groups, and results are reported as the means ± standard error of means (SEMs). All analyses used GraphPad Prism (Version 8) and SPSS (Version 17).

## 3 Results

### 3.1 Description of Clinical Characteristics of the Sample

The clinical characteristics of the participants are listed in Tables 1, 2, 3, 4, 5. In the five‐part analysis, frequent cough and breathlessness were more common in the severe and very severe obstructive ventilation dysfunction group. The clinical characteristics of the total participants and the participants with COPD‐SQ scores ≥ 16 according to the severity of spirometric abnormality are presented in Tables 1 and 3, respectively; participants with severe and very severe obstructive ventilation dysfunction were older, were predominantly male, and had lower SpO_2_ and quicker resting heart rates. The clinical characteristics of the participants with COPD‐PS scores ≥ 5 and the participants with COPD‐PS scores ≥ 5 and COPD‐SQ scores ≥ 16 according to the severity of spirometric abnormality are presented in Tables 2 and 4, respectively, and participants with severe and very severe obstructive ventilation dysfunction were older and had quicker resting heart rates. The clinical characteristics of the male participants according to the severity of spirometric abnormality are presented in Table 5; participants with severe and very severe obstructive ventilation dysfunction were older and had lower SpO_2_ and quicker resting heart rates.

**TABLE 1:** Demographic characteristics of the study population and univariate logistic regression for the prevalence of severe and very severe lung function.

Variables	FEV1% index level			*p*
	Total (*n* = 7679)	(50% ≤ FEV1% < 70%) (*n* = 6214)	(FEV1% < 50%) (*n* = 1465)	
Age, years	68.54 ± 3.85	68.43 ± 3.85	69.03 ± 3.81	0.000
Male, *n* (%)	7557 (98.4%)	6103 (98.21%)	1454 (99.25%)	0.006
Smoking index, pack‐years	50.98 ± 26.06	51.02 ± 26.10	50.79 ± 25.89	0.753
Exposure to dust/hazardous gases, *n* (%)	1026 (13.4%)	833 (13.4%)	193 (13.2%)	0.815
Symptom				
Frequent cough, *n* (%)	2497 (32.5%)	1938 (31.2%)	559 (38.2%)	
Breathlessness, *n* (%)	4606 (60.0%)	3626 (58.4%)	980 (66.9%)	
Blood pressure				
Systolic blood pressure, mmHg	131.4 ± 13.79	131.5 ± 13.72	131.0 ± 14.06	0.152
Diastolic blood pressure, mmHg	79.50 ± 9.36	79.48 ± 9.25	79.58 ± 9.84	0.714
SpO_2_, %	96.43 ± 6.48	96.53 ± 6.12	96.02 ± 7.83	0.008
Resting heart rate, times/min	75.30 ± 9.90	75.02 ± 9.97	76.48 ± 9.51	0.000
Family history of respiratory disease	894 (11.6%)	719 (11.6%)	175 (11.9%)	0.687
COPD‐PS, score	4.60 ± 1.15	4.52 ± 1.09	4.92 ± 1.34	
COPD‐SQ, score	21.02 ± 4.11	20.74 ± 3.98	22.20 ± 4.45	
Lung function				
FEV1, L	1.80 ± 0.47	2.13 ± 0.24	1.20 ± 0.50	
FVC, L	2.68 ± 0.59	2.81 ± 0.51	2.13 ± 0.60	
FEV1/FVC%	67.39 ± 12.45	69.95 ± 10.11	56.54 ± 15.27	

*Note:* Data are presented as mean ± SD or *n* ± %.

Abbreviations: COPD‐PS, COPD population screening; COPD‐SQ, COPD screening questionnaire; FEV1, forced expiratory volume in 1 s; FVC, forced vital capacity; SpO_2_, peripheral capillary oxygen saturation.

**TABLE 2:** Demographic characteristics of the study population with COPD‐PS scores ≥ 5 and univariate logistic regression for the prevalence of severe and very severe lung function.

Variables	FEV1% index level			*p*
	Total (*n* = 3076)	(50% ≤ FEV1% < 70%) (*n* = 2347)	(FEV1% < 50%) (*n* = 729)	
Age, years	68.63 ± 3.86	68.41 ± 3.80	69.35 ± 3.96	0.000
Male, *n* (%)	3031 (98.5%)	2309 (98.4%)	722 (99.0%)	0.201
Smoking index, pack‐years	51.63 ± 27.00	51.54 ± 26.58	51.93 ± 28.33	0.753
Exposure to dust/hazardous gases, *n* (%)	629 (20.4%)	481 (20.5%)	148 (20.3%)	0.860
Symptom				
Frequent cough, *n* (%)	1559 (50.7%)	1162 (49.5%)	397 (54.5%)	
Breathlessness, *n* (%)	2385 (77.5%)	1778 (75.8%)	607 (83.3%)	
Blood pressure				
Systolic blood pressure, mmHg	131.7 ± 13.45	131.7 ± 13.20	131.6 ± 14.23	0.879
Diastolic blood pressure, mmHg	79.12 ± 9.46	78.95 ± 9.30	79.67 ± 9.94	0.075
SpO_2_, %	95.95 ± 7.10	96.09 ± 6.85	95.51 ± 7.86	0.055
Resting heart rate, times/min	75.72 ± 9.61	75.10 ± 9.39	76.86 ± 9.67	0.000
Family history of respiratory disease	410 (13.3%)	310 (13.2%)	100 (13.7%)	0.724
COPD‐PS, score	5.72 ± 0.91	5.60 ± 0.80	5.99 ± 1.07	
COPD‐SQ, score	22.76 ± 4.67	22.36 ± 4.61	24.03 ± 4.63	
Lung function				
FEV1, L	1.74 ± 0.49	1.92 ± 0.32	1.16 ± 0.51	
FVC, L	2.65 ± 0.60	2.81 ± 0.50	2.13 ± 0.59	
FEV1/FVC%	65.79 ± 13.14	69.30 ± 10.25	54.47 ± 14.94	

*Note:* Data are presented as mean ± SD or *n* ± %.

Abbreviations: COPD‐PS, COPD population screening; COPD‐SQ, COPD screening questionnaire; FEV1, forced expiratory volume in 1 s; FVC, forced vital capacity; SpO_2_, peripheral capillary oxygen saturation.

**TABLE 3:** Demographic characteristics of the study population with COPD‐SQ scores ≥ 16 and univariate logistic regression for the prevalence of severe and very severe lung function.

Variables	FEV1% index level			*p*
	Total (*n* = 7516)	(50% ≤ FEV1% < 70%) (*n* = 6070)	(FEV1% < 50%) (*n* = 1446)	
Age, years	68.60 ± 3.85	68.49 ± 3.85	69.06 ± 3.82	0.000
Male, *n* (%)	7398 (98.4%)	5963 (98.2%)	1435 (99.2%)	0.005
Smoking index, pack‐years	50.90 ± 25.99	50.96 ± 26.07	50.63 ± 25.70	0.659
Exposure to dust/hazardous gases, *n* (%)	946 (12.6%)	755 (12.4%)	191 (13.2%)	0.968
Symptom				
Frequent cough, *n* (%)	2496 (33.2%)	1937 (31.9%)	559 (38.7%)	
Breathlessness, *n* (%)	4592 (61.1%)	3616 (59.6%)	976 (67.5%)	
Blood pressure				
Systolic blood pressure, mmHg	131.4 ± 13.78	131.5 ± 13.72	131.0 ± 14.02	0.240
Diastolic blood pressure, mmHg	79.50 ± 9.29	79.49 ± 9.18	79.52 ± 9.77	0.898
SpO_2_, %	96.40 ± 6.54	96.49 ± 6.18	95.99 ± 7.88	0.010
Resting heart rate, times/min	75.33 ± 9.93	75.05 ± 10.00	76.50 ± 9.53	0.000
Family history of respiratory disease	864 (11.5%)	695 (11.4%)	169 (11.7%)	0.799
COPD‐PS, score	4.59 ± 1.16	4.49 ± 1.06	4.92 ± 1.35	
COPD‐SQ, score	21.17 ± 4.01	20.90 ± 3.87	22.31 ± 4.38	
Lung function				
FEV1, L	1.80 ± 0.47	1.94 ± 0.32	1.19 ± 0.49	
FVC, L	2.68 ± 0.60	2.82 ± 0.51	2.13 ± 0.59	
FEV1/FVC%	67.27 ± 12.46	69.88 ± 10.12	56.28 ± 15.11	

*Note:* Data are presented as mean ± SD or *n* ± %.

Abbreviations: COPD‐PS, COPD population screening; COPD‐SQ, COPD screening questionnaire; FEV1, forced expiratory volume in 1 s; FVC, forced vital capacity; SpO_2_, peripheral capillary oxygen saturation.

**TABLE 4:** Demographic characteristics of the study population with COPD‐PS scores ≥ 5 and COPD‐SQ scores ≥ 16 and univariate logistic regression for the prevalence of severe and very severe lung function.

Variables	FEV1% index level			*p*
	Total (*n* = 2915)	(50% ≤ FEV1% < 70%) (*n* = 2205)	(FEV1% < 50%) (*n* = 710)	
Age, years	68.77 ± 3.86	68.57 ± 3.80	69.41 ± 3.98	0.000
Male, *n* (%)	2870 (98.5%)	2167 (98.3%)	703 (99.0%)	0.171
Smoking index, pack‐years	51.45 ± 26.90	51.39 ± 26.52	51.65 ± 28.05	0.826
Exposure to dust/hazardous gases, *n* (%)	563 (19.3%)	436 (19.8%)	127 (17.9%)	0.142
Symptom				
Frequent cough, *n* (%)	1559 (53.5%)	1162 (52.7%)	397 (55.9%)	
Breathlessness, *n* (%)	2367 (81.2%)	1764 (80.0%)	603 (84.9%)	
Blood pressure				
Systolic blood pressure, mmHg	131.6 ± 13.40	131.6 ± 13.16	131.7 ± 14.14	0.865
Diastolic blood pressure, mmHg	79.07 ± 9.28	78.92 ± 9.105	79.55 ± 9.81	0.119
SpO_2_, %	95.84 ± 7.26	95.97 ± 7.02	95.43 ± 7.94	0.086
Resting heart rate, times/min	75.81 ± 9.66	75.46 ± 9.62	76.90 ± 9.71	0.001
Family history of respiratory disease	453 (15.5%)	327 (14.8%)	126 (17.7%)	0.062
COPD‐PS, score	5.75 ± 0.92	5.67 ± 0.85	6.01 ± 1.07	
COPD‐SQ, score	23.25 ± 4.28	22.91 ± 4.19	24.30 ± 4.39	
Lung function				
FEV1, L	1.73 ± 0.50	1.92 ± 0.32	1.15 ± 0.50	
FVC, L	2.65 ± 0.60	2.81 ± 0.50	2.13 ± 0.58	
FEV1/FVC%	65.37 ± 13.16	69.07 ± 10.26	53.89 ± 14.49	

*Note:* Data are presented as mean ± SD or *n* ± %.

Abbreviations: COPD‐PS, COPD population screening; COPD‐SQ, COPD screening questionnaire; FEV1, forced expiratory volume in 1 s; FVC, forced vital capacity; SpO_2_, peripheral capillary oxygen saturation.

**TABLE 5:** Demographic characteristics of the male population and univariate logistic regression for the prevalence of severe and very severe lung function.

Variables	FEV1% index level			*p*
	Total (*n* = 7557)	(50% ≤ FEV1% < 70%) (*n* = 6103)	(FEV1% < 50%) (*n* = 1454)	
Age, years	68.55 ± 3.84	68.44 ± 3.84	69.03 ± 3.81	0.000
Smoking index, pack‐years	51.04 ± 26.07	51.08 ± 26.09	50.86 ± 25.96	0.769
Exposure to dust/hazardous gases, *n* (%)	1009 (13.4%)	817 (13.4%)	192 (13.2%)	0.855
Symptom				
Frequent cough, *n* (%)	2430 (32.2%)	1878 (30.8%)	552 (38.0%)	
Breathlessness, *n* (%)	4504 (59.6%)	3533 (58.2%)	971 (66.8%)	
Blood pressure				
Systolic blood pressure, mmHg	131.5 ± 13.74	131.7 ± 13.65	131.0 ± 14.11	0.085
Diastolic blood pressure, mmHg	79.51 ± 9.39	79.49 ± 9.28	79.60 ± 9.86	0.680
SpO_2_, %	96.41 ± 6.52	96.55 ± 5.93	96.01 ± 7.86	0.042
Resting heart rate, times/min	75.24 ± 9.86	74.93 ± 9.91	76.51 ± 9.52	0.000
Family history of respiratory disease	886 (11.7%)	695 (11.4%)	191 (13.1%)	0.063
COPD‐PS, score	4.60 ± 1.15	4.53 ± 1.08	4.92 ± 1.35	
COPD‐SQ, score	21.01 ± 4.12	20.73 ± 3.98	22.20 ± 4.45	
Lung function				
FEV1, L	1.81 ± 0.46	1.95 ± 0.31	1.20 ± 0.50	
FVC, L	2.70 ± 0.59	2.83 ± 0.50	2.13 ± 0.60	
FEV1/FVC%	67.28 ± 12.44	69.85 ± 10.08	56.51 ± 15.28	

*Note:* Data are presented as mean ± SD or *n* ± %.

Abbreviations: COPD‐PS, COPD population screening; COPD‐SQ, COPD screening questionnaire; FEV1, forced expiratory volume in 1 s; FVC, forced vital capacity; SpO_2_, peripheral capillary oxygen saturation.

Variables with *p*‐values < 0.05 in the univariate analyses and anthropometric variables were included in the following multivariate logistic regression analysis to evaluate the relationship between anthropometric variables and lung function in a severe smoking community population with ventilatory dysfunction.

### 3.2 Anthropometric Variables in Different Groups

**FIGURE 2:**
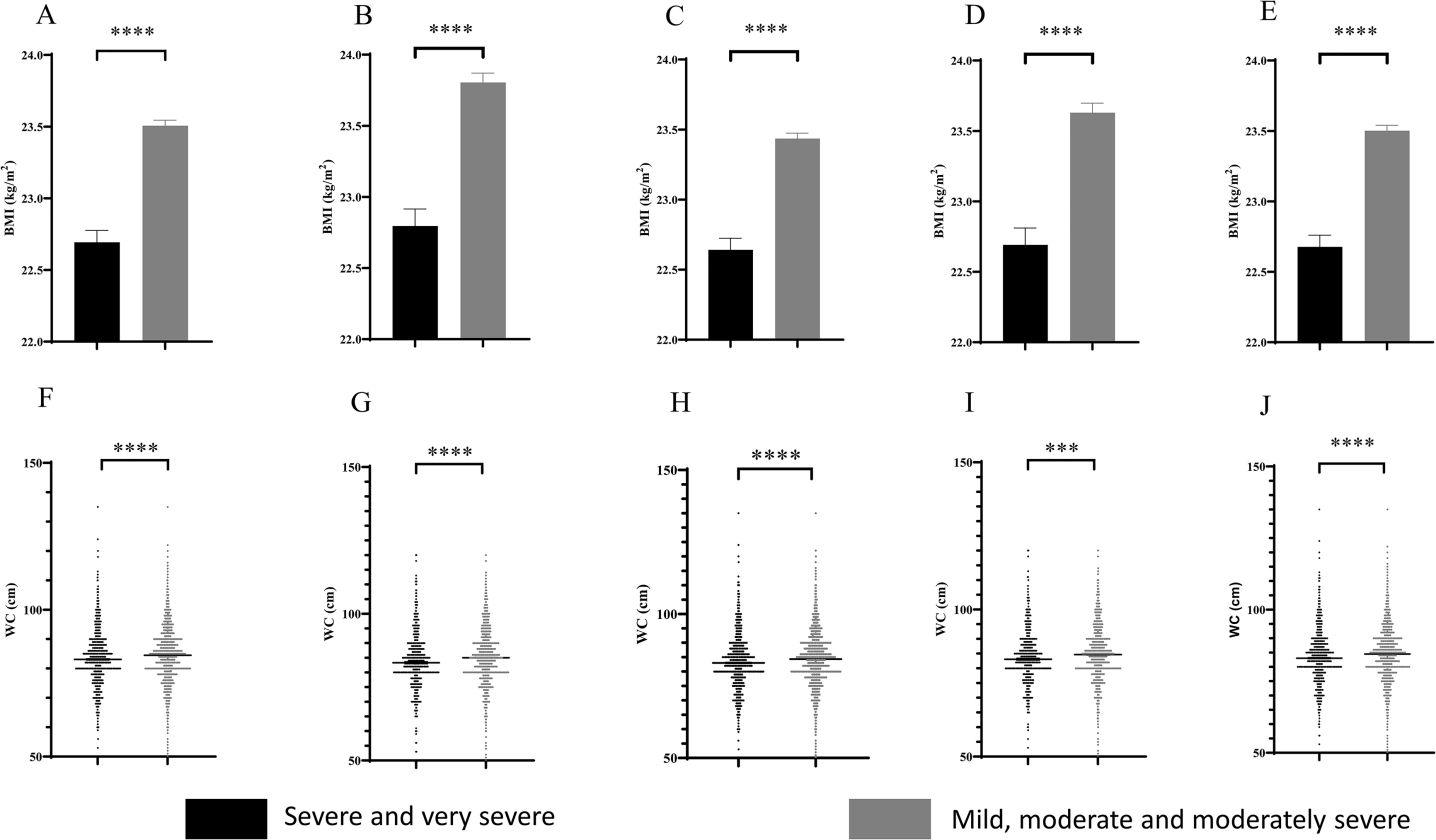
BMI of severe smokers (A). BMI of severe smokers with PS ≥ 5 (B). BMI of severe smokers with SQ ≥ 16 (C). BMI of severe smokers with PS ≥ 5 and SQ ≥ 16 (D). BMI of male severe smokers (E). WC of severe smokers (F). WC of severe smokers with PS ≥ 5 (G). WC of severe smokers with SQ ≥ 16 (H). WC of severe smokers with PS ≥ 5 and SQ ≥ 16 (I). WC of male severe smokers (J). ***p < 0.001, ****p < 0.0001.

**FIGURE 3:**
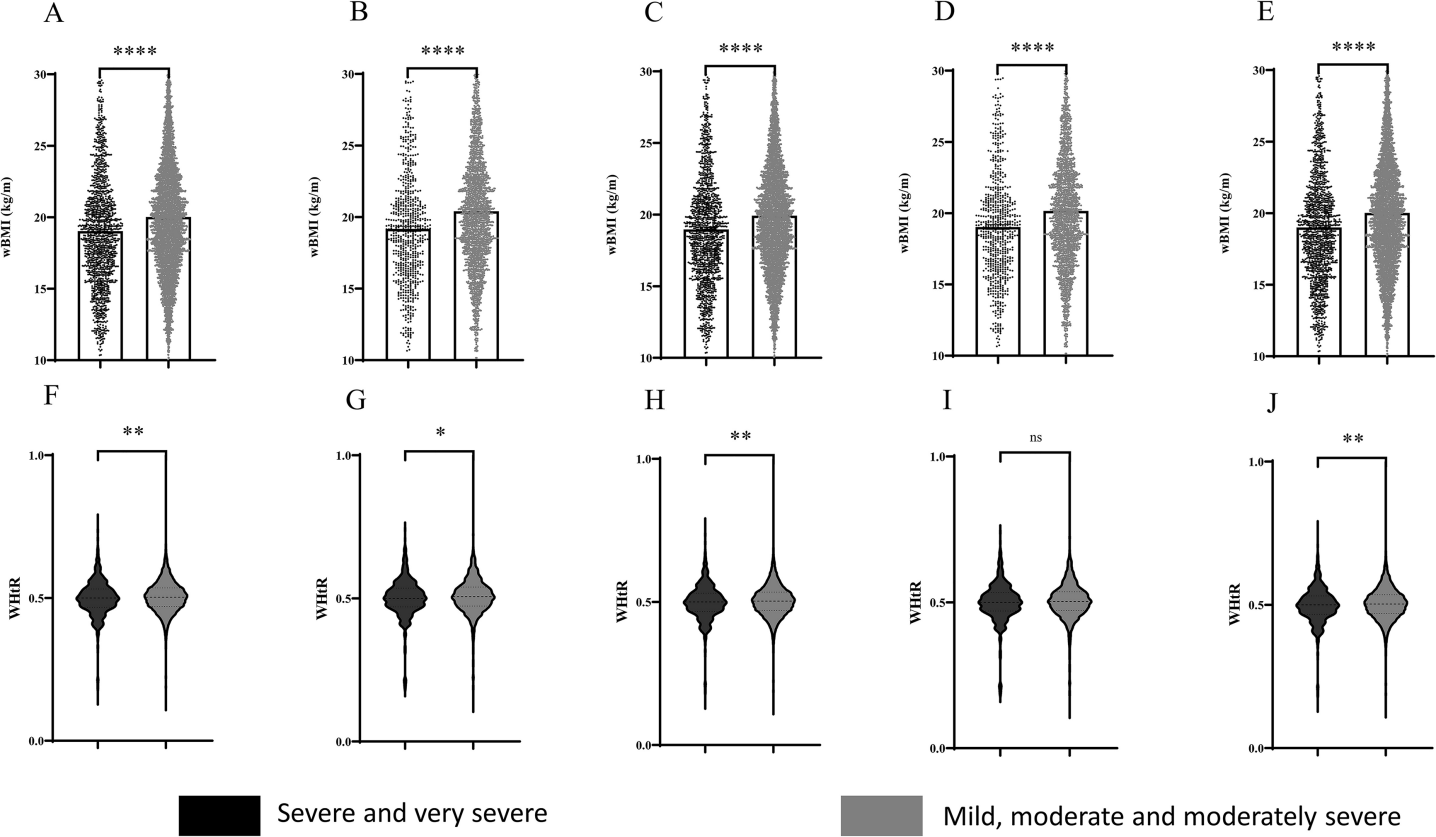
wBMI of severe smokers (A). wBMI of severe smokers with PS ≥ 5 (B). wBMI of severe smokers with SQ ≥ 16 (C). wBMI of severe smokers with PS ≥ 5 and SQ ≥ 16 (D). wBMI of male severe smokers (E). WHtR of severe smokers (F). WHtR of severe smokers with PS ≥ 5 (G). WHtR of severe smokers with SQ ≥ 16 (H). WHtR of severe smokers with PS ≥ 5 and SQ ≥ 16 (I). WHtR of male severe smokers (J). ns, not significant; *p < 0.05; **p < 0.01; ****p < 0.0001.

BMI levels were higher in the individuals with mild, moderate, and moderately severe obstructive ventilation dysfunction than in the individuals with severe and very severe obstructive ventilation dysfunction in all groups (*p* < 0.0001) (Figure 2A–E). WC levels were higher in the individuals with mild, moderate, and moderately severe obstructive ventilation dysfunction than in the individuals with severe and very severe obstructive ventilation dysfunction in all groups (*p* < 0.001, *p* < 0.0001) (Figure 2F–J). wBMI levels were higher in the individuals with mild, moderate, and moderately severe obstructive ventilation dysfunction than in the individuals with severe and very severe obstructive ventilation dysfunction in all groups (*p* < 0.0001) (Figure 3A–E). WHtR levels were higher in the individuals with mild, moderate, and moderately severe obstructive ventilation dysfunction than in the individuals with severe and very severe obstructive ventilation dysfunction in all groups except for those with COPD‐PS scores ≥ 5 and COPD‐SQ scores ≥ 16 (*p* < 0.01, *p* < 0.05) (Figure 3F–J).

### 3.3 Multivariate Analyses

To adjust for confounding effects, significant differences between populations were from the univariate analyses to evaluate patient characteristics, and variables that had two‐sided *p*‐values < 0.05 in the univariate analyses and anthropometric variables were included in the multivariate logistic regression analysis.

**FIGURE 4:**
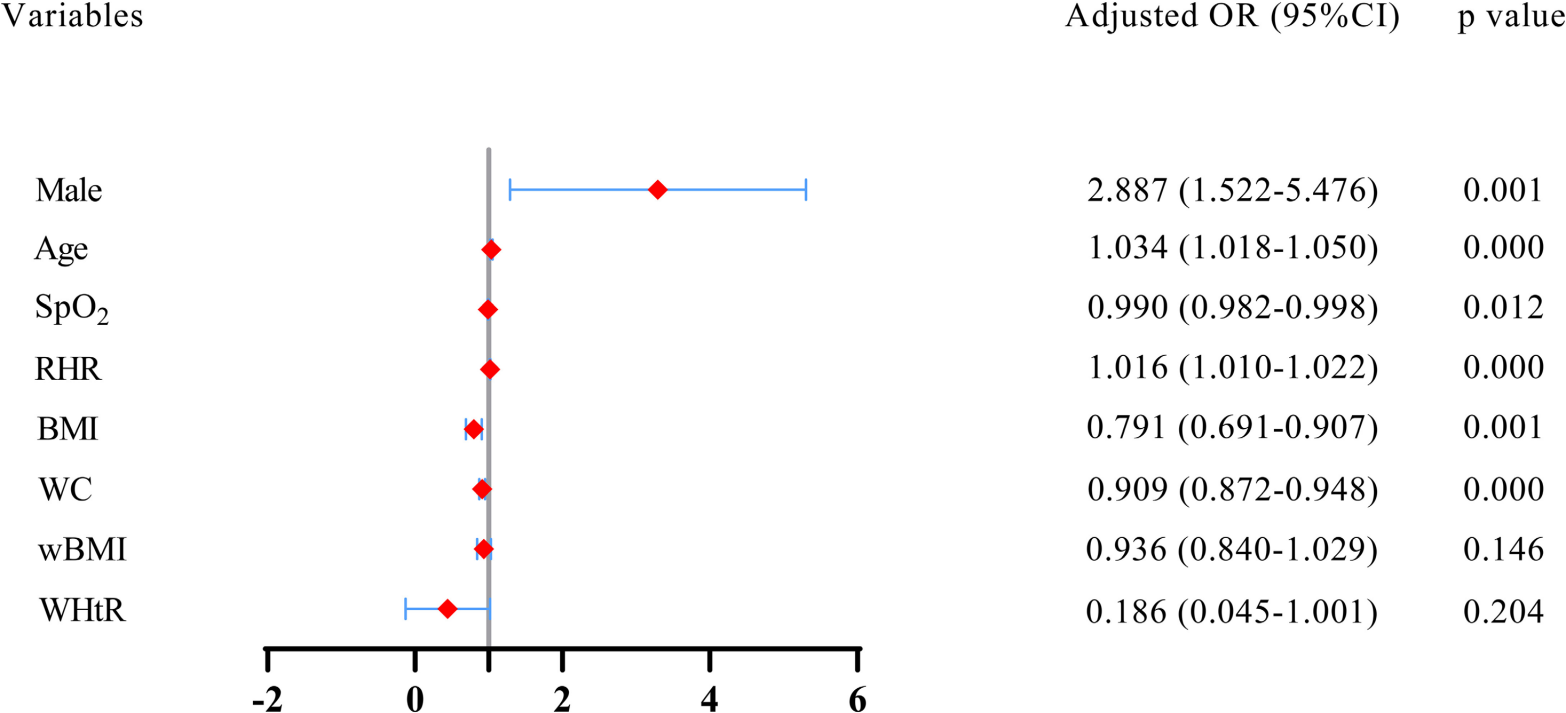
Multivariate logistics regression analysis of the study population. BMI, body mass index; OR, odds ratios; RHR, resting heart rate; SpO_2_, peripheral capillary oxygen saturation; wBMI, waist body mass index; WC, waist circumference; WHtR, waist‐to‐height ratio.

**FIGURE 5:**
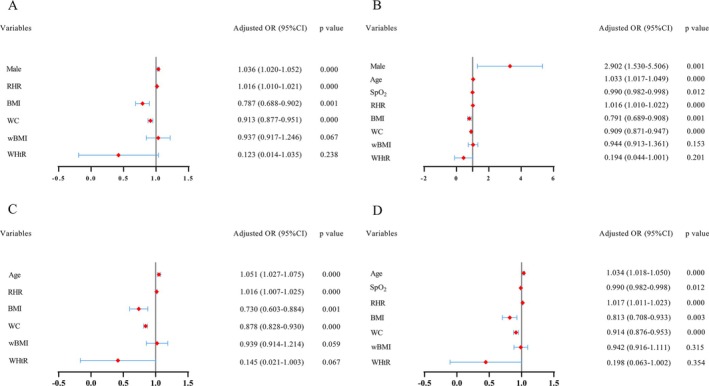
Multivariate logistics regression analysis of the study population with COPD‐PS scores ≥ 5 (A). Multivariate logistics regression analysis of the study population with COPD‐SQ scores ≥ 16 (B). Multivariate logistics regression analysis of the study population with COPD‐PS scores ≥ 5 and COPD‐SQ scores ≥ 16 (C). Multivariate logistics regression analysis of the male population (D). BMI, body mass index; OR, odds ratios; RHR, resting heart rate; SpO_2_, peripheral capillary oxygen saturation; wBMI, waist body mass index; WC, waist circumference; WHtR, waist‐to‐height ratio.

As shown in Figures 4 and 5, when FEV1% < 80%, only BMI and WC in anthropometric variables were identified as independent factors for predicting FEV1% < 50% in the multivariate analysis.

Figures 4 and 5 show the results of the adjusted analyses of BMI when FEV1% < 80% and FEV1% < 50%, respectively, in a severe smoking community population. Severe smokers with a one‐unit increase in BMI had 0.791 times lower odds of severe and very severe obstructive ventilation dysfunction than subjects with mild, moderate, and moderately severe obstructive ventilation dysfunction. In those with COPD‐PS ≥ 5, severe smokers with a one‐unit increase in BMI had 0.787 times lower odds of severe and very severe obstructive ventilation dysfunction than subjects with mild, moderate, and moderately severe obstructive ventilation dysfunction. In those with COPD‐SQ ≥ 16, severe smokers with a one‐unit increase in BMI had 0.791 times lower odds of severe and very severe obstructive ventilation dysfunction than subjects with mild, moderate, and moderately severe obstructive ventilation dysfunction. In those with COPD‐PS ≥ 5 and COPD‐SQ ≥ 16, severe smokers with a one‐unit increase in BMI had 0.730 times lower odds of severe and very severe obstructive ventilation dysfunction than subjects with mild, moderate and moderately severe obstructive ventilation dysfunction. Male severe smokers with a one‐unit increase in BMI had 0.813 times lower odds of severe and very severe obstructive ventilation dysfunction than subjects with mild, moderate and moderately severe obstructive ventilation dysfunction.

Figures 4 and 5 show the results of the adjusted analyses of WC when FEV1% < 80% and FEV1% < 50%, respectively, in a severe smoking community population. Severe smokers with a one‐unit increase in WC had 0.909 times lower odds of severe and very severe obstructive ventilation dysfunction than subjects with mild, moderate, and moderately severe obstructive ventilation dysfunction. In those with COPD‐PS ≥ 5, severe smokers with a one‐unit increase in WC had 0.813 times lower odds of severe and very severe obstructive ventilation dysfunction than subjects with mild, moderate, and moderately severe obstructive ventilation dysfunction. In those with COPD‐SQ ≥ 16, severe smokers with a one‐unit increase in WC had 0.909 times lower odds of severe and very severe obstructive ventilation dysfunction than subjects with mild, moderate, and moderately severe obstructive ventilation dysfunction. In those with COPD‐PS ≥ 5 and COPD‐SQ ≥ 16, severe smokers with a one‐unit increase in WC had 0.878 times lower odds of severe and very severe obstructive ventilation dysfunction than subjects with mild, moderate, and moderately severe obstructive ventilation dysfunction. Male severe smokers with a one‐unit increase in WC had 0.914 times lower odds of severe and very severe obstructive ventilation dysfunction than subjects with mild, moderate, and moderately severe obstructive ventilation dysfunction.

## 4 Discussion

The World Health Organization estimated that 65 million individuals worldwide have mild to severe COPD, and the prevalence is rising [4], resulting in a considerable medical and economic burden. However, there are large numbers with underlying COPD remaining undiagnosed, especially in rural areas. Moreover, Wang et al. [5] found that risk factors for COPD included smoking exposure of 20 pack‐years or more. Therefore, it is of great significance to screen for COPD in a severe smoking community population. Because previous studies have shown that the degree of obesity is strongly associated with COPD, exploring the relationship between anthropometric variables and lung function in severe smokers is of great significance for the management of chronic lung diseases.

COPD‐PS was designed for patients attending 12 primary care settings in the United States, and the AUC of the logistic analysis model was 0.89. When the optimal cut‐off value of the questionnaire was 5, the positive predictive value was 86.4% [6]. Kobayashi et al. [7] studied the application value of the questionnaire in primary medical institutions in Japan and found that the AUC was 0.71 when the optimal cut‐off value was 5 points. Han [8] used the COPD‐PS questionnaire survey for clinic patients and gained satisfying screening efficiency (AUC = 0.84). The COPD‐PS questionnaire is simple and easy to operate, but it has few items, which affects the screening efficiency. Common risk factors, such as dust particles and biomass fuel exposure, were not included. Zhou et al. [3] found that when the optimal cut‐off value was 16 in COPD‐SQ, the sensitivity, specificity, and AUC of the logistic regression model were 73%, 78%, and 0.83, respectively, and the sensitivity, specificity, and AUC of the validation cohort model were 61%, 85%, and 0.81, respectively. COPD‐SQ is the first COPD screening questionnaire designed by Chinese scholars for the Chinese population, with a good prospect for application. Therefore, it is essential to select the population with COPD‐PS scores ≥ 5 and COPD‐SQ scores ≥ 16 among severe smokers for the management of the population who are at risk of COPD.

COPD can cause reduced dietary intake, digestive and absorption dysfunction, and reduced protein and fat synthesis, leaving patients in a state of high decomposition and high metabolism. Malnutrition is one of the most critical and common comorbidities in patients with COPD. Data consistently show that the prevalence of weight loss in COPD is 25%–40% [9]. Malnutrition in COPD patients is often manifested by weight loss and skeletal muscle loss. Low weight was associated with decreased lung function, prolonged hospitalization times and length of stay, and poor survival. Vibhuti et al. [10] found that a positive correlation existed between BMI and FEV1% in AECOPD. In GOLD Grade 4, Kirsch et al. [11] found an almost linear decline in healthcare expenditures with increasing BMI. However, in addition to the previously reported malnutrition, COPD patients, especially those in the stable phase, also suffer from overnutrition. Zhou et al. [12] also found that low BMI and obese subjects had lower FEV1 after adjustment. Tang et al. [13] discovered that being underweight and severely obese were associated with reduced lung function, and slight obesity was shown to be a protective factor for lung function in people at risk of COPD and those with preserved ratio impaired spirometry.

In anthropometric variables, BMI eliminates the influence of height on weight and can reflect the nutritional status of the human body well. A study found that BMI, BMI, WC and WHtR all have a statistically significant positive correlation with fat mass percentage [14]. Foumani et al. [15] found that WC was not observed to impact lung function in this study, but it was a predictive factor for COPD severity in patients. The risk of incident COPD was positively associated with increasing WC among Chinese adults of both sexes in a study [16]. Popović‐Grle et al. [17] failed to prove its importance in correlation with functional lung capacity in a selected COPD population. A finding suggested that visceral fat accumulation may increase obstructive pulmonary disease development in young males and accelerate the decline of pulmonary function in older females [18]. Emami Ardestani et al. [19] found a statistically significant relationship between FEV1% predicted and BMI and WC. Sato et al. [20] found a statistically significant relationship between FEV1% predicted and BMI and WC. Individuals with severe COPD are often at risk of undernutrition. When stratified according to WC, underweight was associated with a higher mortality of chronic inflammatory airway disease [21]. Undernutrition can worsen COPD and other comorbidities and can be an independent predictor of morbidity and functional decline, resulting in increased healthcare consumption and increased risk of death; and anthropometry was used to assess undernutrition, including weight, BMI, waist, hip and upper arm circumference.

Some scholars have also studied the relationship between WHtR and lung function. Molina‐Luque et al. [22] discovered that WHtR ≥ 0.55 was significantly related to increased lung age. A study found that obesity was associated with self‐reported asthma and pulmonary function limitations, and the association was stronger when the measurement of obesity was based on body fat percentage or WHtR, compared to BMI. There was a higher self‐reported asthma risk among obese women, according to the WHtR [23]. Zeng et al. [24] found that WHtR was negatively associated with lung function in rural Chinese adults. In a Mediterranean population of smokers without respiratory symptoms, abdominal obesity, evaluated not only by BMI and WC but also by WHtR, is inversely associated with lung function [25]. He et al. held that abdominal obesity indices were negatively associated with lung function, and the associations may be partly mediated by systemic inflammation [26]. Ishikawa et al. [27] have also observed that simple measures such as WHtR were sufficient to detect the association of body composition with pulmonary function reduction. However, few scholars have studied the relationship between WHtR and lung function in COPD. Few studies have investigated the relationship between wBMI and lung function in COPD.

The aforementioned studies and this study examined the relationship between anthropometric variables and lung function. However, this study investigated the population according to the risk of COPD assessed by the two questionnaires and, for the first time, compared the OR values of BMI, WC, BMI and WHtR in a severe smoking community population with severe and very severe obstructive ventilation dysfunction.

Recently, the “obesity paradox” has been paid more attention to. We refer to the obesity paradox when particular chronic diseases reviewed hereunder exhibit an interesting “paradoxical” protective association between BMI and clinical outcomes. Many longitudinal studies have shown a positive correlation between obesity and mortality, and several cross‐sectional and retrospective studies have shown a negative correlation between BMI and mortality in most cases when BMI is < 30. However, after BMI exceeded 30, there was a clear positive association between obesity and all‐cause mortality. In addition, conditions such as heart failure, coronary heart disease, hypertension, diabetes, chronic kidney disease, infection, hepatitis C, COVID‐19, and COPD are common causes of death. Studies have found that COPD and obesity often coexist, and there are complex interaction mechanisms. A multicenter, prospective cohort study on the genetic epidemiology of COPD found that the risk factor for COPD was abnormal lung function rather than obesity, and there was no conclusive evidence that obesity and COPD were causal [28]. The impact of BMI on the lung function of the COPD population in China was first studied by Wu et al. [29], who proposed the so‐called “obesity paradox” in Asian COPD patients. However, some studies have shown that obesity is beneficial in many situations. In a study, the impact of obesity on short‐term and long‐term outcomes after COPD exacerbations was assessed. This was a prospective, randomized, unblinded clinical trial that showed that obesity was associated with reduced mortality within 1.5 years after COPD exacerbation. Although obese patients had a higher incidence of comorbidities, multivariate regression analysis showed that they had a lower mortality rate within 1 year [30]. The obesity paradox has been postulated to occur in three ways: emphysema, the influence of body components, or a spurious conclusion. In the future, the concept of the “obesity paradox” may be altered. Therefore, it makes sense to explore the relationship between different anthropometric variables and lung function in a severe smoking community population with ventilatory dysfunction.

Our results revealed that lung function in the severe smoking community population was associated with anthropometric variables like BMI, WC, wBMI, and WHtR. When FEV1% < 80%, only BMI and WC were identified as independent factors for predicting FEV1% < 50% in the multivariate analysis. Moreover, the OR for predicting FEV1% < 50% decreased as the included population was at risk of COPD. Meanwhile, lung function in a male severe smoking community population was less associated with anthropometric variables than with the overall population. However, wBMI and WHtR were not identified as independent factors. This study is of great significance for the assessment of anthropometric variables in a severe smoking community population at high risk of COPD.

There are some restrictions in this research. First, we performed only pulmonary function screening, not COPD diagnosis tests. Airflow obstruction (FEV1/FVC < 0.7 post‐bronchodilation) is a criterion for diagnosing COPD. However, there is no relevant reference investigating an impact on the results. Second, the population was all from the community and not representative. The relationship between anthropometric variables and lung function in the severe smoking community population still needs to be further studied. Third, lung imaging conditions were not obtained because this study was part of an extensive population survey. However, smoking has a significant impact on emphysema and fibrosis in the severe smoking community population. Further studies will be undertaken if lung imaging conditions become available.

## 5 Conclusions

Anthropometric variables are associated with lung function in the severe smoking community population, especially when the lung function index is poor. When FEV1% < 80%, appropriate anthropometric variable gains, like BMI and WC, appear to benefit the individual. Unfortunately, decreased lung function caused by smoking is not managed well in the community as chronic diseases. In the future, mass screening of anthropometric variables and lung function can facilitate the identification of underlying COPD and effective community management.

## Author Contributions

All authors read the final version of the manuscript. Study design and manuscript writing: Tiantian Cen. Data collection, analysis, and interpretation: Tiantian Cen, Zekai Cen, and Xuan Chen. Manuscript editing and approval of the final version: Yiming Yu, Shanshan Wang, and Hongying Ma.

## Ethics Statement

The First Affiliated Hospital of Ningbo University's ethical committee granted consent for this study (approval number KY20221208). We attest that the survey was carried out considering the Helsinki Declaration of 1964 and any subsequent changes.

## Conflicts of Interest

The authors declare no conflicts of interest.

## Data Availability

The datasets used and/or analyzed during this study are available from the corresponding author upon reasonable request.
